# Supplementary material for: A Review of Anthropometric Measurements for Saudi Adults and Elderly, Directions for Future Work and Recommendations to Establish Saudi Guidelines in Line with the Saudi 2030 Vision
Source: Healthcare (Basel). 2023 Jul 8;11(14):1982. doi: 10.3390/healthcare11141982 (PMC10378928; doi:10.3390/healthcare11141982)
Supplement: Supplementary file 1 [file healthcare-11-01982-s001.zip › healthcare-2369653-supplementary.pdf]

Supplementary Material

A review of anthropometric measurements for Saudi adults and elderly, directions for future work and recommendations to establish Saudi guidelines in line with the Saudi 2030 Vision

Essra A. Noorwali*, Abeer M. Aljaadi

*** Correspondence:** Corresponding Author: eanoorwali@uqu.edu.sa

Table S1. Saudi adult/elderly studies assessing anthropometric measurements.

No.	Author, Year (reference)	Region/City	Population age	Sample n	Anthropometrics assessed	Anthropometrics assessment definition	Comments
					**Adult studies**		
**1.**	Al-Haboubi, 1992 [1]	Dhahran	20–59 years	408	19 body dimensions	Compared to other nationalities	Study only included male participants. Anthropometric measurement method not mentioned
**2.**	Al-Shammari et al. 1994 [2]	Riyadh	women	1385	BMI		No access to the paper-abstract only available
**3.**	Rasheed et al. 1994 [3]	Dammam	18–25 years	222	Weight, height and triceps skinfold measurements	Several references	Anthropometric measurement method was mentioned
**4.**	Khalid 1995 [4]	Southern SA	16–60 years	905	Weight, height, mid-upper arm circumference, and skinfold thickness		No access to the paper-abstract only. Compared between low and high-altitude residents
**5.**	Kordy and El-gamal 1995 [5]	Jeddah	≥16 years	1037	Weight, height	WHO	Anthropometric measurement method mentioned
**6.**	Al-Nuaim et al. 1996 [6]	5 regions	15–95 years	13,177	Weight, height, BMI	WHO	Anthropometric measurement method mentioned
**7.**	Al-Nuaim et al. 1997 [7]	5 regions	≥20 years	10,651	Weight, height, BMI	WHO	Anthropometric measurement method mentioned

**8.**	Ahmed and ElZubair 1997 [8]	Al-Khobar	Saudis	1160	Weight, height, waist and hip circumferences, BMI, waist/hip ratio		No access to the paper-abstract only available
**9.**	Soyannwo et al. 1998 [9]	Buraidah, Gassim	Saudi children and adults	6,044	BMI		No access to the paper-abstract only available
**10.**	Rasheed 1998 [10]	Al-Khobar	women	144	Weight, height, BMI	NHANES-I	Anthropometric measurement method mentioned
**11.**	Al-Shammari et al. 2001 [11]	Riyadh	>17 years	2,927	Weight, height, BMI	WHO	Anthropometric measurement method was mentioned.
**12.**	Alsaif et al. 2002 [12]	Three regions in SA	30–70 years	3,271	Weight, height, and waist and hip circumferences, BMI	Cutoffs based on [13–15]	Anthropometric measurement method was mentioned. Compared the prevalence of overweight and obesity between KSA and other populations
**13.**	Ardawi et al. 2002 [16]	Jeddah	20–69 years	1426	Weight, height, BMI, waist-to-hip ratio	Not mentioned	Anthropometric measurement method not mentioned
**14.**	Al-Harithy 2003 [17]	Jeddah	19–30 years	65	Weight, height, waist and hip circumference. BMI	Not mentioned	Anthropometric measurement method not mentioned
**15.**	Akbar et al. 2003 [18]	Jeddah	Adults	541	Weight, height, BMI	Compared to non-Saudis	Anthropometric measurement method not mentioned
**16.**	Al-Harithy 2004 [19]	Jeddah	20–75 years	122	Weight, height, waist, and hip circumferences, BMI	Not mentioned	Anthropometric measurement method not mentioned
**17.**	Ogbeide et al. 2004 [20]	AlKharj	Adults	994	Weight, height, BMI	Not mentioned	Anthropometric measurement method not mentioned
**18.**	Al-Ajlan and Al-Mehdi 2005 [21]	Riyadh	adults	474	BMI	Not mentioned	Anthropometric measurement method not mentioned
**19.**	Al-Nozha et al. 2005 [22]	Rural and urban	30–70 years	17,232	Weight, Height, BMI	WHO/NHLBI	Anthropometric measurement method mentioned. Compared the prevalence of obesity between KSA and other gulf countries

**20.**	Al-Qahtani et al. 2005 [23]	North	soldiers 20–60 years	2,250	Weight, Height, BMI, waist and hip circumference, waist-hip ratio	WHO	Anthropometric measurement method mentioned
**21.**	Al-Nozha et al. 2005 [24]	Rural and urban	30–70 years	17,293	Weight, height, waist circumference, BMI	NCEP-(ATP) III	Anthropometric measurement method mentioned
**22.**	Alissa et al. 2006 [25]	Jeddah	males	140	Weight, height, BMI	WHO	Anthropometric measurement method not mentioned
**23.**	Ismail et al. 2006 [26]	Hofuf	20–80 years	243	Weight, height, BMI	Not available	Anthropometric measurement method not mentioned
**24.**	Al-Sultan and Al-Elq 2006 [27]	AlKhobar	adults	89	Weight, height, BMI, waist and hip circumference	Not available	Anthropometric measurement method mentioned
**25.**	Al-Qahtani et al. 2006 [28]	60 km to the west of Hafr Al-Batin	18–59 years	2577	Weight, height, BMI, waist circumference	NCEP-ATPIII and IDF criteria	Anthropometric measurement method mentioned
**26.**	Khalid 2007 [29]	Abha	18–60 years	438	Weight, height, BMI, waist circumference	WHO	Anthropometric measurement method mentioned
**27.**	Al-Nozha et al. 2007 [30]	Urban and rural	30–70 years	17,395	Weight, height, BMI and waist circumference	Based on several references	Anthropometric measurement method mentioned. Study measured physical activity levels
**28.**	Al-Daghri et al. 2007 [31]	Riyadh	Adults	308	Weight, height, BMI, waist and hip circumference	Not mentioned	Anthropometric measurement method mentioned
**29.**	Al-Baghli et al. 2008 [32]	Eastern	≥30 years	195,874	Weight, height, BMI	NHLBI	Anthropometric measurement method mentioned
**30.**	Yar 2008 [33]	Dammam	university students	123	Weight, height, BMI	WHO	Anthropometric measurement method mentioned
**31.**	Al-Gelban 2008 [34]	Abha	21–25 years	456	Weight, height, BMI	Not mentioned	Anthropometric measurement method not mentioned
**32.**	Al-Habdan et al. 2009 [35]	Al-Khobar	female	6,400	Weight, height, BMI	Not mentioned	Anthropometric measurement method mentioned
**33.**	Taha et al. 2009 [36]	Taif and Alula	20–30 years	646	26 anthropometric measures	Compared to Malaysian men	Anthropometric measurement method mentioned
**34.**	Al-Rethaiaa et al. 2010 [37]	Qassim	18–24 years	357	Weight, height BMI, body fat percent and visceral fat level	NIH and other references	Anthropometric measurement method mentioned

**35.**	Al-Qauhiz 2010 [38]	Eastern	university students	799	Weight, height, BMI		No access to the paper-abstract only available
**36.**	Bahathiq 2010 [39]	Makkah	university students	240	Weight, height, BMI, waist circumference	Based on [40]	Anthropometric measurement method mentioned
**37.**	Yar 2010 [41]	Dammam	university students	231	Weight, height, BMI, waist circumference, waist-to-hip ratio, waist-to-stature ratio	Based on Chinese and Korean cutoffs [42–44]	Anthropometric measurement method mentioned
**38.**	Al-Hamdan et al. 2010 [45]	All regions	15–64 years	4758	Weight, height, BMI	WHO	Anthropometric measurement method mentioned
**39.**	Khalil et al. 2011 [46]	Qassim	adults	177	Weight, height, BMI, waist-hip ratio	WHO	Anthropometric measurement method mentioned. The study assessed the relationship between BMI and skin conditions
**40.**	Al-Ajlan 2011 [47]	Riyadh	18–35 years	333	weight, height, waist and hip circumferences, BMI	WHO	Anthropometric measurement method mentioned
**41.**	Shaheen et al. 2011 [48]	Riyadh	18–26 years	69	Weight, height, waist and abdominal circumference, waist-hip-ratio, BMI	WHO	Anthropometric measurement method mentioned
**42.**	Azeem 2011 [49]	Eastern	University students	30	Weight, height, BMI	Not available	Anthropometric measurement method mentioned
**43.**	Alkadi and Alissa 2011 [50]	Jeddah	university students	127	Weight, height, waist and hip circumference, BMI, waist-hip-ratio	WHO and other references	Anthropometric measurement method mentioned
**44.**	Al-Ajlan 2012 [51]	Riyadh	college students	474	Weight, height, waist circumference and hip circumference, BMI	Not available	Anthropometric measurement method mentioned
**45.**	Allam et al. 2012 [52]	Madinah	university students	194	Weight, height, waist and hip circumference, BMI, waist-to-hip ratio	NIH	Anthropometric measurement method not mentioned
**46.**	Warsy et al. 2012 [53]	Riyadh	18–75 years females	126	Weight, height, BMI, waist and hip circumference, waist-hip ratio	WHO	Anthropometric measurement method mentioned briefly

**47.**	Habib 2012 [54]	Riyadh	Adults	229	weight, height, BMI, WHR, fat mass, lean body mass, and muscle mass	Not mentioned	Anthropometric measurement method mentioned
**48.**	Horaib et al. 2013 [55]	5 regions	military	10,229	Weight, height, waist and hip circumference	WHO	Anthropometric measurement method mentioned
**49.**	AlDokhi and Habib 2013 [56]	Riyadh	18–72 years	411	Weight, height, BMI, % body fat	WHO	Anthropometric measurement method mentioned
**50.**	Al-Daghri et al. 2013 [57]	Riyadh	19–60 years	185	Weight, height, BMI, waist and hip circumference	IDF	Anthropometric measurement method mentioned briefly.
**51.**	Saeed and Al-Hamdan 2013 [58]	All regions	15–64 years	4758	Weight, height, BMI, waist and hip circumference	WHO	Anthropometric measurement method mentioned. This study was a national survey.
**52.**	Habib 2013 [59]	Riyadh	18–72 years	530	Weight, height, BMI, body composition	WHO	Anthropometric measurement method mentioned
**53.**	Bin Ammar and Al-Holy 2013 [60]	Hail	15–50 years	80	Weight, height, BMI	WHO	Anthropometric measurement method mentioned
**54.**	Al-Otaibi et al. 2013 [61]	Al-Ahsa	≥18 years	368	Weight, height, BMI	WHO	Anthropometric measurement method mentioned
**55.**	Mohamed 2013 [62]	Abha	women	360	Weight, height, BMI	Not mentioned	Anthropometric measurement method mentioned
**56.**	Naji et al. 2013 [63]	Majmaah	university students	303	Weight, height, BMI, waist circumference	WHO	Anthropometric measurement method mentioned
**57.**	Habib 2013 [64]	Riyadh	≥18	428	Weight, BMI, protein mass, fat mass, %body fat	WHO	Anthropometric measurement method mentioned
**58.**	Ahmed et al. 2014 [65]	Hail	adults	5000	Weight, height, BMI	WHO	Anthropometric measurement method not mentioned. The study assessed the prevalence of obesity in Hail
**59.**	Basulaiman et al. 2014 [66]	All regions	≥15 years	10,735	Weight, height, BMI	Not mentioned	Anthropometric measurement method mentioned briefly. Data were from the Saudi health interview survey. The study identified risk

							factors of hypercholesteremia. Risk increased in obese participants
**60.**	Al-Daghri et al. 2014 [67]	Riyadh	18–70 years	9164	Weight, height, waist and hip circumference, BMI	Used cutoffs for metabolic syndrome ATP III	Anthropometric measurement method briefly mentioned
**61.**	Al-Daghri et al. 2014 [68]	Riyadh	adults	927	Weight, height, BMI, waist and hip circumference	Not mentioned	Anthropometric measurement method mentioned
**62.**	Faris et al. 2014 [69]	Hail	College students	314	Weight, height, BMI, body fat%, waist-to-hip ratio	WHO and other references	Anthropometric measurement method mentioned
**63.**	Alharbi et al. 2014 [70]	Riyadh	adults	367	Weight, height, BMI	Not mentioned	Anthropometric measurement method mentioned
**64.**	Memish et al. 2014 [71]	All regions	≥15 years	10,735	Weight, height, BMI	WHO	Anthropometric measurement method not mentioned
**65.**	Al-Daghri et al. 2014 [72]	Riyadh	university students	1878	Weight, height, waist and hip circumference, % body fat	Not mentioned	Anthropometric measurement method mentioned
**66.**	Alrashdan et al. 2014 [73]	Riyadh	18–25 years	150	32 body measurements	Compared to Belgium, Chinese, Turkish, USA, Netherland, Brazil, Australia	Anthropometric measurement method mentioned
**67.**	Banoar et al. 2014 [74]	Hail	18–30 years	237	Weight, height, BMI, % body fat	WHO	Anthropometric measurement method mentioned
**68.**	Amin et al. 2014 [75]	Al-Hassa	24–63 years	691	Weight, height, BMI, waist circumference	WHO	Anthropometric measurement method mentioned
**69.**	El Bcheraoui et al. 2014 [76]	All regions	≥15 years	10,735	Weight, height, BMI	Not mentioned	Anthropometric measurement method mentioned
**70.**	Brocato et al. 2015 [77]	Jeddah	adults	2686	Weight, height, BMI	Not mentioned	Anthropometric measurement method mentioned
**71.**	Al-Daghri et al. 2015 [78]	Riyadh	adults and children	1,385	Weight, height, BMI, waist circumference	Based on Cole et al. [79]	Anthropometric measurement method mentioned

**72.**	AlDokhi 2015 [80]	Riyadh	18–72 years	461	Weight, height, BMI, protein mass, muscle mass, fat mass, %body fat	Not mentioned	Anthropometric measurement method mentioned
**73.**	Al-Shammari et al. 2015 [81]	Hail	≥18 years	237	Weight, height, BMI, body composition	WHO	Anthropometric measurement method mentioned
**74.**	Fatima and Rakib 2015 [82]	Hail	Adults	528	Weight, height, BMI, waist circumference	WHO	Anthropometric measurement method was mentioned
**75.**	Moradi-Lakeh et al. 2015 [83]	All regions	≥ 15 years	10,735	Weight, height, BMI	Not mentioned	Anthropometric measurement method not mentioned. Data were from the Saudi Health Interview Survey. A representative sample.
**76.**	Alshahwan et al. 2015 [84]	Riyadh	≥21 years	45	Weight, height, BMI, waist and hip circumference	Not mentioned	Anthropometric measurement method mentioned
**77.**	Memish et al. 2015 [85]	5 regions	≥ 20 years	1485	Weight, height, BMI, waist circumference	IDF	Anthropometric measurement method not mentioned
**78.**	Zeidan et al. 2016 [86]	Al-Madinah	women aged 20–36	279	Weight, height, BMI	Not mentioned	Anthropometric measurement method mentioned
**79.**	El Bcheraoui et al. 2016 [87]	All regions	≥15 years	10,735	Weight, height, BMI	Not mentioned	Anthropometric measurement method not mentioned
**80.**	Aboel-Fetoh et al. 2016 [88]	Arar	≥15 years	401	Weight, height, BMI		No access to the paper- just the abstract
**81.**	Hamouda 2016 [89]	Al-Leith	17–30 years	100	Weight, height, BMI, waist, hip, neck circumference, %body fat	Not mentioned	Anthropometric measurement method mentioned
**82.**	AlKhalaf et al. 2016 [90]	All regions	>18 years	12,744	Weight, height, BMI, waist and hip circumference	European Caucasian cutoffs	This is an abstract
**83.**	Mahassni and Bashanfar 2016 [91]	Jeddah	18–28 years	112	Weight, height, BMI, waist and hip circumference, waist-to-hip ratio	Based on several references	Anthropometric measurement method was mentioned
**84.**	Mahmoud and Ncibi 2016 [92]	Jazan	19–21 years	182	Weight, height, BMI	Based on references	Kinanthropometry method

**85.**	Brahim et al. 2016 [93]	Riyadh	18–20 years	293	Weight, height, BMI, waist circumference	CDC	Anthropometric measurement method was mentioned-compared to American population
**86.**	Mansour 2016 [94]	Aseer	19–30 years	900	12 anthropometric dimensions	Compared to Turkish and Iranian populations	Anthropometric measurement method not mentioned
**87.**	Abdel-Salam 2016 [95]	Al-Jouf	19–25 years	340	Weight, height, BMI, waist circumference	WHO	Anthropometric measurement method mentioned
**88.**	Abd El-Al 2016 [96]	Wadi Aldawaser	20–60 years	310	weight, height, waist circumference, hip circumference	WHO	Anthropometric measurement method mentioned
**89.**	Bahijri et al. 2016 [97]	Jeddah	≥18 years	1420	Weight, height, BMI, waist and hip circumference,	Based on several references	Anthropometric measurement method mentioned
**90.**	Habib et al. 2017 [98]	Riyadh	University students	211	Weight, height, BMI	Cutoffs mentioned but no reference	Anthropometric measurement method mentioned
**91.**	Khabaz et al. 2017 [99]	Jeddah	University students	116	Weight, height, BMI	Cutoffs mentioned but no reference	Anthropometric measurement method not mentioned
**92.**	Alahmari et al. 2017 [100]	Abha	20–74 years	116	Weight, height, BMI, hand length, forearm circumference	Cutoffs mentioned but no reference	Anthropometric measurement method mentioned
**93.**	Kaddam et al. 2017 [101]	3 regions	6–62 years	6,139	Weight, height, BMI	Cutoffs mentioned but no reference	Anthropometric measurement method mentioned
**94.**	Alghamdi 2017 [102]	Jeddah	Adults	140	Weight, height, BMI, waist and hip circumference	WHO	Anthropometric measurement method mentioned
**95.**	Abulmeaty et al. 2017 [103]	Riyadh	18–50 years	390	weight, height, waist, hip, mid-arm and wrist circumference, triceps skin-fold thickness	Framingham score	Anthropometric measurement method mentioned
**96.**	Albawardi et al. 2017 [104]	Riyadh	18–60 years	420	Weight, height, BMI	WHO	Anthropometric measurement method was mentioned

**97.**	Azzeh et al. 2017 [105]	Makkah, Jeddah, Al-Taif	18–60 years	2548	Weight, height, BMI, waist circumference and body composition	Based on several references	Anthropometric measurement method mentioned
**98.**	AlQahtani et al. 2017 [106]	Riyadh	≥18 years	200	Weight, height, BMI	WHO	Anthropometric measurement method mentioned
**99.**	Altowaijri et al. 2018 [107]	Tabuk	20–40 years	350	Weight, height, BMI	WHO	Anthropometric measurement method mentioned
**100.**	Al-Ghamdi et al. 2018 [108]	Al-Kharj	≥18 years	1019	Weight, height, BMI, waist circumference	WHO	Anthropometric measurement method mentioned
**101.**	Basuliman 2018 [109]	Riyadh	19–26 years	93	18 dimensions	WHO	Anthropometric measurement method mentioned
**102.**	Al-Rashdan et al. 2018 [110]	Riyadh	25–60 years	154	15 measurements	Not mentioned	Anthropometric measurement method mentioned
**103.**	Latif and Rafique 2018 [111]	Dammam	Female adults	106	Weight, height, BMI, Waist Circumference, Waist-Hip Ratio, Body Density, Body Adiposity Index , % Body fat	WHO and several references	Anthropometric measurement method mentioned
**104.**	Al-Rubean et al. 2018 [112]	All regions	≥18 years	12,126	Weight, height, waist and hip circumference	IDF/ NCEP ATP III	Anthropometric measurement method mentioned. The study measured the prevalence of metabolic syndrome and their risk factors in Saudi adults.
**105.**	Gutierrez et al. 2018 [113]	Tabuk	≥18 years	432	Weight, height, BMI	CDC	Anthropometric measurement method mentioned
**106.**	Aldossari et al. 2018 [114]	AlKharj	≥18 years	381	Weight, height, BMI, waist circumference	WHO/ADA	Anthropometric measurement method mentioned
**107.**	Alsheef et al. 2018 [115]	Riyadh	Men	269	Weight, height, BMI, waist and hip circumference, waist-to-height ratio	WHO	Anthropometric measurement method not mentioned
**108.**	Alzahrani et al. 2019 [116]	AlKharj	≥18 years	638	Weight, height, BMI, waist circumference	Based on several references	Anthropometric measurement method mentioned
**109.**	AlMuzaini et al. 2019 [117]	Madina	18–69 years	395	Weight, height, BMI,	WHO	Anthropometric measurement method mentioned
**110.**	Rafique et al. 2019 [118]	Dammam	18–21 years	744	Weight, height, BMI	WHO	Anthropometric measurement method mentioned

**111.**	AlQuaiz et al. 2019 [119]	Riyadh	30–75 years	2997	Weight, height, BMI, waist circumference	WHO	Anthropometric measurement method mentioned
**112.**	Al-Qahtani 2019 [120]	Southwestern	Adults	1681	Weight, height, BMI	WHO	Anthropometric measurement method mentioned
**113.**	Alamri 2019 [121]	Tabuk	University students	450	Weight, height, BMI	WHO	Anthropometric measurement method mentioned
**114.**	Wahabi et al. 2019 [122]	Riyadh	Postpartum women	115	Weight, height, BMI	WHO	Anthropometric measurement method not mentioned
**115.**	AlNasser et al. 2019 [123]	Riyadh	≥18 years	240	Weight, height, BMI, waist circumference	WHO	Anthropometric measurement method mentioned
**116.**	Dhaifullah et al. 2019 [124]	Riyadh	21–35 years	308	Weight, height, BMI	WHO	Anthropometric measurement method mentioned
**117.**	Tedla et al. 2019 [125]	Abha	20–23 years	240	Weight, height, upper, lower limb length, trunk length	Not mentioned	Anthropometric measurement method mentioned. The study formulated normative values of the forward reach test and lateral reach test and their correlation with anthropometrics.
**118.**	Al-Raddadi et al. 2019 [126]	Jeddah	≥18 years	1419	Weight, height, BMI	Several references	Anthropometric measurement method referred to a reference
**119.**	Al-Bassam et al. 2019 [127]	Riyadh	18–70 years	623	Weight, height, BMI, neck and waist circumference, % body fat	IDF	Anthropometric measurement method mentioned
**120.**	Binobeaid et al. 2019 [128]	Riyadh	20–80 years	63	Weight, height, BMI	WHO	Anthropometric measurement method mentioned
**121.**	Header et al. 2019 [129]	Western	19–45 years	100	Weight, height, BMI, waist, hip, arm circumference, body composition	Cutoffs mentioned but no reference	Anthropometric measurement method mentioned
**122.**	Farhat et al. 2019 [130]	Riyadh	30–95 years	1702	Weight, height, BMI	WHO	Anthropometric measurement method mentioned
**123.**	El-Metwally et al. 2019 [131]	Al-Kharj	Adults	1031	Weight, height, BMI, waist circumference	WHO	Anthropometric measurement method mentioned
**124.**	Alfaris et al. 2020 [132]	Riyadh	Women	456	Weight, height, BMI,	Not mentioned	Anthropometric measurement method mentioned

					circumferences of waist, hip, and mid-upper arm, skinfold thickness at triceps and body composition		
**125.**	Alkahtani et al. 2020 [133]	Riyadh	20–80 years	363	Weight, height, BMI, body composition	Several references	Anthropometric measurement method mentioned
**126.**	Amer et al. 2020 [134]	Riyadh	25–60 years	180	Weight, height, BMI, waist circumference	Cutoffs mentioned but no reference	Anthropometric measurement method not mentioned
**127.**	Al-Hanawi et al. 2020 [135]	All regions	>15 years	7746	Weight, height, BMI	Continuous BMI variable	Anthropometric measurement method referred to a reference. Representative sample from the Saudi Health Interview survey
**128.**	Syed et al. 2020 [136]	Jazan	18–26	416	Weight, height, BMI	NHLBI/NIH	Anthropometric measurement method not mentioned
**129.**	Thomas et al. 2020 [137]	Majmaah	18–40 years	307	Weight, height, BMI, waist circumference	WHO	Anthropometric measurement method mentioned
**130.**	Mumena et al. 2020 [138]	Madinah	University students	190	Weight, height, BMI, waist circumference	WHO	Anthropometric measurement method mentioned
**131.**	Shaaban and Ali 2020 [139]	Makkah	18–20 years	145	Height, length of the upper and lower limb, chest depth and circumference	Compared to Egyptian adults	Anthropometric measurement method mentioned
**132.**	Mosli et al. 2020 [140]	Jeddah/Makkah	≥18 years	3925	Weight, height, BMI	Based on references	Anthropometric measurement method mentioned
**133.**	Naguib et al. 2020 [141]	Riyadh	University females	550	Weight, height, BMI, body composition, waist , hip circumference, triceps skinfolds	Based on references	Anthropometric measurement method mentioned
**134.**	Toriola and Onagbiye 2020 [142]	Riyadh	≥18 years	183	Weight, height, BMI	Cole et al	Anthropometric measurement method mentioned
**135.**	Alahmari et al. 2020 [143]	Abha	University students	52	Weight, height, waist and thigh circumference, skinfold thickness, thigh length, lower-limb length	Not mentioned	Anthropometric measurement method mentioned

**136.**	Alzeidan et al. 2020 [144]	Riyadh	Adults	3063	Weight, height, BMI, waist circumference	WHO and other references	Anthropometric measurement method mentioned. The study found that waist-to-height ratio using global cutoff showed good diagnostic performance for cardiovascular risk factors and metabolic syndrome.
**137.**	Muaidi and Ahsan 2020 [145]	Dammam	University students	550	Weight, height, BMI, waist, hip circumference	Not mentioned	Anthropometric measurement method mentioned
**138.**	Sami et al. 2020 [146]	Taif	Children, adolescents, adults	1602	Weight, height, BMI	WHO	Anthropometric measurement method mentioned
**139.**	Aldossari et al. 2020 [147]	AlKharj	≥18 years	1003	Weight, height, BMI, waist circumference	Not mentioned	Anthropometric measurement method not mentioned
**140.**	AlShamiri et al. 2020 [148]	Riyadh	≥18 years	200	Weight, height, BMI, waist circumference, waist-to-height ratio	Based on several references	Anthropometric measurement method mentioned
**141.**	Bahijri et al. 2020 [149]	Jeddah	18–55 years	98	Weight, height, BMI, waist circumference	WHO	Anthropometric measurement method mentioned
**142.**	Al-Musharaf et al. 2021 [150]	Riyadh	19–30 years	355	Weight, height, BMI, waist, hip circumference	WHO	Anthropometric measurement method mentioned
**143.**	Al-Musharaf et al. 2021 [151]	Riyadh	19–30 years	297	Weight, height, BMI	Not mentioned	Anthropometric measurement method mentioned
**144.**	Al-Musharaf et al. 2021 [152]	Riyadh	19–30 years	355	Weight, height, BMI, waist and hip circumference, waist/hip ratio	WHO	Anthropometric measurement method mentioned
**145.**	Al-quaiz et al. 2021 [153]	Riyadh	30–75 years	2997	Weight, height, BMI	WHO	Anthropometric measurement method mentioned
**146.**	Aldossari et al. 2021 [154]	AlKharj	≥18 years	1019	Weight, height, BMI	WHO	Anthropometric measurement method mentioned
				**Elderly**			

**147.**	Abolfotouh et al. 2001 [155]	Abha	≥65 years	810	Waist circumference, Waist-to-hip ratio	WHO and several references	Anthropometric measurement method was mentioned
**148.**	Al-Othaimeen et al. 2007 [156]	All regions	Children, adolescents, adults and elderly were included	19,598	Weight, height, BMI, skin fold thickness and mid-arm circumference	Based on several reverences	Anthropometric measurement method not mentioned
**149.**	Al-Zahrani et al. 2016 [157]	Jeddah	60–90 years	152	Weight, height, BMI, calf circumference	MNA-SF	Anthropometric measurement method mentioned
**150.**	Alhamdan et al. 2020 [158]	Riyadh	≥60 years	2045	Body composition	MNA	Anthropometric measurement method mentioned
**151.**	AlTurki et al. 2021	Riyadh	≥60 years	38	Weight, height, BMI	MNA	Anthropometric measurement method not mentioned

BMI: Body mass index, CDC: Centers for Disease Control, IDF: International Diabetes Federation, IOTF: International Obesity Task force, ISO: International Organization for Standardization, LBW: low birth weight, MNA-SF: Mini Nutritional Assessment-Short form, NCHS: National Center for Health Statistics, NHANESI: National Health and Nutrition Examinations Survey I, NCEP-ATP III: National Cholesterol Education Program's Adult Treatment Panel, NIH: National institute of health, NHLBI: : National institute of health, national heart, Lung and blood institute SA: Saudi Arabia, WHO: World Health Organization

Table S2. The WHO guidelines and recommendations for developing anthropometric cutoff points (waist circumference and waist-hip ratio) [13]

1. **The data are representative of all population groups** (with respect to age, sex, social class, and concurrent diseases) in countries from all regions
2. **Data collected include anthropometric measures (of both central adiposity and BMI**) and at least three risk factors (e.g., blood pressure, blood glucose and cholesterol)
3. **Use standardized methods for measurement** of waist circumference and other anthropometric indicators
4. **Do not use self-reported data of anthropometrics**
5. **Preference of using longitudinal data** than cross-sectional which may be confounded by effects of existing disease and its diagnosis and treatment on risk status or associations
6. **Use absolute risks rather than relative risks** when comparing risk factor or disease levels at different levels of waist circumference or waist–hip ratio. Absolute risks are more relevant from a policy perspective

7. **Linking waist circumference or waist-hip ratio with BMI** might contribute to the development of composite indices for use with individuals and the community.
8. **Receiver operating characteristic (ROC) curves method in determining cutoff points have potential limitations** including differences in cut-off points based on differences in population characteristics (e.g. average body size or disease prevalence). The choice of method will depend on the potential uses of the derived cut-off points and health-relevant policy considerations.

1. Al-Haboubi, M.H. Anthropometry for a mix of different populations. *Appl. Ergon.* **1992**, *23*, doi:10.1016/0003-6870(92)90224-J.

2. Al-Shammari, S.A.; Khoja, T.A.; Al-Maatouq, M.A.; Al-Nuaim, L.A. High prevalence of clinical obesity among Saudi females: A prospective, cross-sectional study in the Riyadh region. *J. Trop. Med. Hyg.* **1994**, *97*.

3. Rasheed, P.; Abou-Hozaifa, B.M.; Khan, A. Obesity among young saudi female adults: A prevalence study on medical and nursing students. *Public Health* **1994**, *108*, doi:10.1016/S0033-3506(94)80008-1.

4. Khalid, M. Anthropometric comparison between high- and low-altitude saudi arabians. *Ann. Hum. Biol.* **1995**, *22*, doi:10.1080/03014469500004122.

5. Kordy, M.N.; el-gamal, F.M. A study of pattern of body mass index (BMI) and prevalence of obesity in a Saudi population. *Asia. Pac. J. Public Health* **1995**, *8*, doi:10.1177/101053959500800201.

6. Al-Nuaim, A.R.; Al-Rubeaan, K.; Al-Mazrou, Y.; Al-Attas, O.; Al-Daghari, N.; Khoja, T. High prevalence of overweight and obesity in Saudi Arabia. *Int. J. Obes.* **1996**, *20*.

7. Al-Nuaim, A.A.; Bamgboye, E.A.; Al-Rubeaan, K.A.; Al-Mazrou, Y. Overweight and obesity in Saudi Arabian adult population, role of socio- demographic variables. *J. Community Health* **1997**, *22*, doi:10.1023/A:1025177108996.

8. Ahmed, G.; Zubier., E. Waist/hip ratio as a predictor of diabetes mellitus and hypertension in primary health care centres, Al-Khobar, Saudi Arabia. *Bull. High Inst. Public Heal.* **1997**, *27*, 571–580.

9. Soyannwo, M.A.; Kurashi, N.Y.; Gadallah, M.; Hams, J.; el-Essawi, O.; Khan, N.A.; Singh, R.G.; Alamri, A.; Beyari, T.H. Body

mass index (BMI) in the Saudi population of Gassim. *Afr. J. Med. Med. Sci.* **1998**, *27*.

10. Rasheed, P. Perception of body weight and self-reported eating and exercise behaviour among obese and non-obese women in Saudi Arabia. *Public Health* **1998**, *112*, doi:10.1016/S0033-3506(98)00288-1.

11. Al-Shammari, S.A.; Khoja, T.; Gad, A. Community-based study of obesity among children and adults in Riyadh, Saudi Arabia. *Food Nutr. Bull.* **2001**, *22*, doi:10.1177/156482650102200209.

12. Alsaif, M.A.; Hakim, I.A.; Harris, R.B.; Alduwaihy, M.; Al-Rubeaan, K.; Al-Nuaim, A.R.; Al-Attas, O.S. Prevalence and risk factors of obesity and overweight in adult Saudi population. *Nutr. Res.* **2002**, *22*, doi:10.1016/S0271-5317(02)00439-6.

13. Clinical guidelines on the identification, evaluation, and treatment of overweight and obesity in adults - The evidence report. *Obes. Res.* **1998**, *6*.

14. Lean, M.E.J.; Han, T.S.; Morrison, C.E. Waist circumference as a measure for indicating need for weight management. *BMJ* **1995**, *311*, doi:10.1136/bmj.311.6998.158.

15. Molarius, A.; Seidell, J.C.; Sans, S.; Tuomilehto, J.; Kuulasmaa, K. Varying sensitivity of waist action levels to identify subjects with overweight or obesity in 19 populations of The WHO MONICA Project. *J. Clin. Epidemiol.* **1999**, *52*, doi:10.1016/S0895-4356(99)00114-6.

16. Ardawi, M.S.M.; Rouzi, A.A.; Qari, M.H.; Dahlawi, F.M.; Al-Raddadi, R.M. Influence of age, sex, folate and vitamin B12 status on plasma homocysteine in Saudis. *Saudi Med. J.* **2002**, *23*.

17. Al-Harithy, R.N. Dehydroepiandrosterone sulfate levels in women. Relationships with body mass index, insulin and glucose levels. *Saudi Med. J.* **2003**, *24*.

18. Akbar, D.H.; Ahmed, M.M.; Algamdi, A.A. Cardiovascular risk factors in Saudi Arabian and non-Saudi Arabian diabetic patients in Saudi Arabia. *East. Mediterr. Heal. J.* **2003**, *9*, doi:10.26719/2003.9.5-6.884.

19. Al-Harithy, R.N. Relationship of leptin concentration to gender, body mass index and age in Saudi adults. *Saudi Med. J.* **2004**, *25*.

20. Ogbeide, D.O.; Karim, A.; Al-Khalifa, I.M.; Siddique, S. Population based study of serum lipid levels in Al-Kharj Health Center, Saudi Arabia. *Saudi Med. J.* **2004**, *25*.

21. Al-Ajlan, A.R.; Mehdi, S.R. Effects and a dose response relationship of physical activity to high density lipoprotein cholesterol and

body mass index among Saudis. *Saudi Med. J.* **2005**, *26*.

22. Al-Nozha, M.M.; Al-Mazrou, Y.Y.; Al-Maatouq, M.A.; Arafah, M.R.; Khalil, M.Z.; Khan, N.B.; Al-Marzouki, K.; Abdullah, M.A.; Al-Kadra, A.H.; Al-Harthi, S.S.; et al. Obesity in Saudi Arabia. *Saudi Med. J.* **2005**, *966*.

23. Al-Qahtani, D.A.; Imtiaz, M.L.; Shareef, M.M. Obesity and cardiovascular risk factors in Saudi adult soldiers. *Saudi Med. J.* **2005**, *26*.

24. Al-Nozha, M.M.; Al-Khadra, A.; Arafah, M.R.; Al-Maatouq, M.A.; Khalil, M.Z.; Khan, N.B.; Al-Mazrou, Y.Y.; Al-Marzouki, K.; Al-Harthi, S.S.; Abdullah, M.; et al. Metabolic syndrome in Saudi Arabia. *Saudi Med. J.* **2005**, *26*.

25. Alissa, E.M.; Bahjri, S.M.; Al-Ama, N.; Ahmed, W.H.; Ferns, G.A.A. High cardiovascular risk in young Saudi males: Cardiovascular risk factors, diet and inflammatory markers. *Clin. Chim. Acta* **2006**, *365*, doi:10.1016/j.cca.2005.09.007.

26. Ismail, A.I.; Al-Abdulwahab, A.H.; Al-Mulhim, A.S. Osteoarthritis of knees and obesity in Eastern Saudi Arabia. *Saudi Med. J.* **2006**, *27*.

27. Al-Sultan, A.I.; Al-Elq, A.H. Leptin levels in normal weight and obese saudi adults. *J. Family Community Med.* **2006**, *13*.

28. Al-Qahtani, D.A.; Imtiaz, M.L.; Saad, O.S.; Hussein, N.M. A comparison of the prevalence of metabolic syndrome in Saudi adult females using two definitions. *Metab. Syndr. Relat. Disord.* **2006**, *4*, doi:10.1089/met.2006.4.204.

29. Khalid, M.E. The prevalence of abdominal obesity and its associated risk factors in married, non-pregnant women born and living in high altitude, southwestern, Saudi Arabia. *Saudi Med. J.* **2007**, *28*.

30. Al-Nozha, M.M.; Al-Hazzaa, H.M.; Arafah, M.R.; Al-Khadra, A.; Al-Mazrou, Y.Y.; Al-Maatouq, M.A.; Khan, N.B.; Al-Marzouki, K.; Al-Harthi, S.S.; Abdullah, M.; et al. Prevalence of physical activity and inactivity among Saudis aged 30-70 years: A population-based cross-sectional study. *Saudi Med. J.* **2007**, *28*.

31. Al-Daghri, N.M.; Al-Attas, O.S.; Al-Rubeaan, K.; Mohieldin, M.; Al-Katari, M.; Jones, A.F.; Kumar, S. Serum leptin and its relation to anthropometric measures of obesity in pre-diabetic Saudis. *Cardiovasc. Diabetol.* **2007**, *6*, doi:10.1186/1475-2840-6-18.

32. Al-Baghli, N.A.; Al-Ghamdi, A.J.; Al-Turki, K.A.; El-Zubaier, A.G.; Al-Ameer, M.; Al-Baghli, F.A. Overweight and obesity in the eastern province of Saudi Arabia. *Saudi Med. J.* **2008**, *29*.

33. Yar, T. Spinal shrinkage as measure of spinal loading in male Saudi university students and its relationship with body mass index.

Saudi Med. J. **2008**, *29*.

34. Al-Gelban, K.S. Dietary habits and exercise practices among the students of a Saudi Teachers' Training College. *Saudi Med. J.* **2008**, *29*.

35. Al-Habdan, I.M.; Sadat-Ali, M.; Al-Muhanna, F.A.; Al-Elq, A.H.; Al-Mulhim, A.A.A. Bone mass measurement using quantitative ultrasound in healthy Saudi women: A cross-sectional screening. *Saudi Med. J.* **2009**, *30*.

36. Taha, Z.; Jomoah, I.M.; Zadry, H.R. A study of anthropometric characteristics between Malaysian and Saudi Arabian males aged 20 to 30 years. *J. Hum. Ergol. (Tokyo).* **2009**, *38*, doi:10.11183/jhe.38.27.

37. Al-Rethaiaa, A.S.; Fahmy, A.E.A.; Al-Shwaiyat, N.M. Obesity and eating habits among college students in Saudi Arabia: A cross sectional study. *Nutr. J.* **2010**, *9*, doi:10.1186/1475-2891-9-39.

38. Al Qauhiz, N.M. Obesity among Saudi Female University Students: Dietary Habits and Health Behaviors. *J. Egypt. Public Health Assoc.* **2010**, *85*.

39. Saeed Bahathiq, A. Relationship of Leptin Hormones with Body Mass Index and Waist Circumference in Saudi Female Population of the Makkah Community~!2009-04-22~!2009-06-16~!2010-07-20~! *Open Obes. J.* **2010**, *2*, doi:10.2174/1876823701002010095.

40. Al-Quaiz, A.J.M. Current concepts in the management of obesity: An evidence based review. *Saudi Med. J.* 2001, *22*.

41. Yar, T. Resting Heart Rate and Its Relationship With General and Abdominal Obesity in Young Male Saudi University. *Pakistan J. Physiol.* **2010**, *6*, 6–13.

42. Lee, S.Y.; Park, H.S.; Kim, D.J.; Han, J.H.; Kim, S.M.; Cho, G.J.; Kim, D.Y.; Kwon, H.S.; Kim, S.R.; Lee, C.B.; et al. Appropriate waist circumference cutoff points for central obesity in Korean adults. *Diabetes Res. Clin. Pract.* **2007**, *75*, doi:10.1016/j.diabres.2006.04.013.

43. Zhou, B.F. Predictive values of body mass index and waist circumference for risk factors of certain related diseases in Chinese adults--study on optimal cut-off points of body mass index and waist circumference in Chinese adults. *Biomed. Environ. Sci.* **2002**, *15*, doi:10.1046/j.1440-6047.11.s8.9.x.

44. Han, T.S.; Sattar, N.; Lean, M. ABC of obesity: Assessment of obesity and its clinical implications. *Br. Med. J.* 2006, *333*.

45. Al-Hamdan, N.; Saeed, A.; Kutbi, A.; Choudhry, A.J.; Nooh, R. Characteristics, risk factors, and treatment practices of known adult

hypertensive patients in saudi arabia. *Int. J. Hypertens.* **2010**, *2010*, doi:10.4061/2010/168739.

46. Khalil, G.M.; Al Shobaili, H.A.; Alzolibani, A.; Al Robaee, A. Relationship between obesity and other risk factors and skin disease among adult Saudi population. *J. Egypt. Public Health Assoc.* **2011**, *86*, doi:10.1097/01.EPX.0000398942.36374.5c.

47. Al-Ajlan, A.R. Lipid profile in relation to anthropometric measurements among college male students in riyadh, saudi arabia: A cross-sectional study. *Int. J. Biomed. Sci.* **2011**, *7*.

48. Shaheen, A.; El-Sobkey, B.; Ibrahim, H. Anthropometric Measurement and Ventilatory Function in Obese and Non-Obese Female College Students. *Middle East J. Sci. Res.* **2011**.

49. Azeem, K. Effect of Twelve Weeks Brisk Walking on Blood Pressure , Body Mass Index , and Anthropometric Circumference of Obese Males. **2011**.

50. Al Kadi, H.; Alissa, E. Prevalence of hyperlipidemia and associated risk factors among healthy young Saudi females:relationship with waist Circumference and body Mass Index. *Endocrinol. Metab. Syndr.* **2011**, *s2*, 2–6, doi:10.4172/2161-1017.s2-001.

51. Abdul Rahman Al-Ajlan Tobacco smoking vs. lipid profile and anthropometric measures: A cross- sectional study among students in the Riyadh College of Health Sciences. *African J. Pharm. Pharmacol.* **2012**, *6*, doi:10.5897/ajpp11.741.

52. Allam, A.R.; Taha, I.M.; Al-Nozha, O.M.; Sultan, I.E. Nutritional and health status of medical students at a university in northwestern Saudi Arabia. *Saudi Med. J.* **2012**, *33*.

53. Warsy, A.; Othman, N.; Habib, Z.; Addar, M.; AlDbass, A.; Alanazi, M. Menopause related blood pressure increase and its relation to anthropometric measurements in Saudi females. *Biosci. Biotechnol. Res. Asia* **2012**, *9*, doi:10.13005/bbra/985.

54. Habib, S.S. Serum resistin levels in patients with type 2 diabetes mellitus and its relationship with body composition. *Saudi Med. J.* **2012**, *33*.

55. Horaib, G. Bin; Al-Khashan, H.I.; Mishriky, A.M.; Selim, M.A.; AlNowaiser, N.; BinSaeed, A.A.; Alawad, A.D.; Al-Asmari, A.K.; AlQumaizi, K. Prevalence of obesity among military personnel in Saudi Arabia and associated risk factors. *Saudi Med. J.* **2013**, *34*.

56. Al Dokhi, L.; Habib, S.S. Assessment of gender differences in body composition and physical fitness scoring in saudi adults by bioelectrical impedance analysis. *Acta Clin. Croat.* **2013**, *52*.

57. Al-Daghri, N.M.; Khan, N.; Alkharfy, K.M.; Al-Attas, O.S.; Alokail, M.S.; Alfawaz, H.A.; Alothman, A.; Vanhoutte, P.M. Selected

dietary nutrients and the prevalence of metabolic syndrome in adult males and females in Saudi Arabia: A pilot study. *Nutrients* **2013**, *5*, doi:10.3390/nu5114587.

58. Saeed, A.A.; Al-Hamdan, N.A. Anthropometric risk factors and predictors of hypertension among Saudi adult population - A national survey. *J. Epidemiol. Glob. Health* **2013**, *3*, doi:10.1016/j.jegh.2013.08.004.

59. Habib, S.S. Body mass index and body fat percentage in assessment of obesity prevalence in saudi adults. *Biomed. Environ. Sci.* **2013**, *26*, doi:10.3967/0895-3988.2013.02.003.

60. Ben-Ammar, A.A.; Al-Holy, M.A. Body image and lifestyle attitudes of female gymnasium users in Saudi Arabia. *Nutr. Food Sci.* **2013**, *43*, doi:10.1108/NFS-10-2012-0110.

61. AL-Otaibi, H.H.; Nassef, S.L.; Raouf, T.A. Body Shape Dissatisfaction, Weight Status and Physical Activity among a Sample University Students in Saudi Arabia. *Food Nutr. Sci.* **2013**, *04*, doi:10.4236/fns.2013.46079.

62. Mohamed, A.Y.I. Assessment of anthropometric measurements and nutrition information of some Saudi women in Abha city. **2013**, *9*, 2252–2260.

63. Naji, E.; Kashoo, F.Z.; Kashoo, M. Prevalence of Obesity and Overweight among Majmaah University Students. *Indian J. Physiother. Occup. Ther. - An Int. J.* **2013**, *7*, doi:10.5958/j.0973-5674.7.4.119.

64. Habib, S.S. Body composition analysis and estimation of physical fitness by scoring grades in Saudi adults. *J. Pak. Med. Assoc.* **2013**, *63*.

65. Ahmed, H.G.; Ginawi, I.A.; Elasbali, A.M.; Ashankyty, I.M.; Al-Hazimi, A.M. Prevalence of obesity in hail region, KSA: In a comprehensive survey. *J. Obes.* 2014, *2014*.

66. Basulaiman, M.; El Bcheraoui, C.; Tuffaha, M.; Robinson, M.; Daoud, F.; Jaber, S.; Mikhitarian, S.; Wilson, S.; Memish, Z.A.; Al Saeedi, M.; et al. Hypercholesterolemia and its associated risk factors-Kingdom of Saudi Arabia, 2013. *Ann. Epidemiol.* **2014**, *24*, doi:10.1016/j.annepidem.2014.08.001.

67. Al-Daghri, N.M.; Alkharfy, K.M.; Al-Attas, O.S.; Khan, N.; Alfawaz, H.A.; Alghanim, S.A.; Al-Yousef, M.A.; Al-Ajlan, A.S.M.; Alokail, M.S. Gender-dependent associations between socioeconomic status and metabolic syndrome: A cross-sectional study in the adult Saudi population. *BMC Cardiovasc. Disord.* **2014**, *14*, doi:10.1186/1471-2261-14-51.

68. Al-Daghri, N.M.; Alkharfy, K.M.; Al-Attas, O.S.; Krishnaswamy, S.; Mohammed, A.K.; Albagha, O.M.; Alenad, A.M.; Chrousos,

G.P.; Alokail, M.S. Association between type 2 diabetes mellitus-related SNP variants and obesity traits in a Saudi population. *Mol. Biol. Rep.* **2014**, *41*, doi:10.1007/s11033-014-3022-z.

69. Faris, "Mo'ez Al-Islam" Assessing Obesity, Body Fatness and Dietary Behaviors among Adult College Students in Hail, Saudi Arabia. *Int. J. Nutr. Food Sci.* **2014**, *3*, doi:10.11648/j.ijnfs.20140302.17.

70. Alharbi, K.K.; Richardson, T.G.; Khan, I.A.; Syed, R.; Mohammed, A.K.; Boustred, C.R.; Gaunt, T.R.; Tamimi, W.; Al-Daghri, N.M.; Day, I.N.M. Influence of adiposity-related genetic markers in a population of Saudi Arabians where other variables influencing obesity may be reduced. *Dis. Markers* **2014**, *2014*, doi:10.1155/2014/758232.

71. Memish, Z.A.; El Bcheraoui, C.E.; Tuffaha, M.; Robinson, M.; Daoud, F.; Jaber, S.; Mikhitarian, S.; Al Saeedi, M.; AlMazroa, M.A.; Mokdad, A.H.; et al. Obesity and associated factors - Kingdom of Saudi Arabia, 2013. *Prev. Chronic Dis.* **2014**, *11*, doi:10.5888/pcd11.140236.

72. Al-Daghri, N.M.; Al-Othman, A.; Al-Attas, O.S.; Alkharfy, K.M.; Alokail, M.S.; Albanyan, A.; Sabico, S.; Chrousos, G.P. Stress and cardiometabolic manifestations among Saudi students entering universities: A cross-sectional observational study. *BMC Public Health* **2014**, *14*, doi:10.1186/1471-2458-14-391.

73. Alrashdan, A.; Alsudairi, L.; Alqaddoumi, A. Anthropometry of Saudi Arabian female college students. In Proceedings of the IIE Annual Conference and Expo 2014; 2014.

74. Banoar, R.; Al-Shammari, E.; Al-Rasihidi, S. Physical activity, dietary fiber and fat intake and anthropometric indices of uni- versity students and staff in Hail city of Saudi Arabia. *Int. J. Rev. Life Sci.* **2014**, *4*, 22–29.

75. Amin, T.T.; Al Sultan, A.I.; Mostafa, O.A.; Darwish, A.A.; Al-Naboli, M.R. Profile of non-communicable disease risk factors among employees at a Saudi University. *Asian Pacific J. Cancer Prev.* **2014**, *15*, doi:10.7314/APJCP.2014.15.18.7897.

76. El Bcheraoui, C.; Basulaiman, M.; Tuffaha, M.; Daoud, F.; Robinson, M.; Jaber, S.; Mikhitarian, S.; Memish, Z.A.; Al Saeedi, M.; AlMazroa, M.A.; et al. Status of the diabetes epidemic in the Kingdom of Saudi Arabia, 2013. *Int. J. Public Health* **2014**, doi:10.1007/s00038-014-0612-4.

77. Brocato, J.; Wu, F.; Chen, Y.; Shamy, M.; Alghamdi, M.A.; Khoder, M.I.; Alkhatim, A.A.; Abdou, M.H.; Costa, M. Association between sleeping hours and cardiometabolic risk factors for metabolic syndrome in a saudi arabian population. *BMJ Open* **2015**, *5*, doi:10.1136/bmjopen-2015-008590.

78. Al-Daghri, N.M.; Aljohani, N.; Al-Attas, O.S.; Krishnaswamy, S.; Alfawaz, H.; Al-Ajlan, A.; Alokail, M.S. Dairy products consumption and serum 25-hydroxyvitamin D level in Saudi children and adults. *Int. J. Clin. Exp. Pathol.* **2015**, *8*.

79. Cole, T.J.; Bellizzi, M.C.; Flegal, K.M.; Dietz, W.H. Establishing a standard definition for child overweight and obesity worldwide: International survey. *Br. Med. J.* **2000**, *320*, doi:10.1136/bmj.320.7244.1240.

80. Al-Dokhi, L. Association of the new index of sarcopenic obesity with physical fitness in healthy Saudi men and women. *Eur. Rev. Med. Pharmacol. Sci.* **2015**, *19*.

81. Al-Shammari, E.; Bano, R.; Al Rashidi, S. Impact of Physical activity and Intake of fiber and fat on the anthropometric indices of university females in Hail city of Saudi Arabia. *Curr. Res. Nutr. Food Sci. J.* **2015**, *3*, doi:10.12944/crnfsj.3.2.04.

82. Fatima, S.; Rakib, N. PREVALENCE OF METABOLIC SYNDROME AND ITS RELATION TO BODY COMPOSITION IN A SAUDI FEMALE POPULATION. *Int. J. Pharm. Ther.* **2015**, 96–102.

83. Moradi-Lakeh, M.; El Bcheraoui, C.; Tuffaha, M.; Daoud, F.; Al Saeedi, M.; Basulaiman, M.; Memish, Z.A.; AlMazroa, M.A.; Al Rabeeah, A.A.; Mokdad, A.H. Self-Rated Health Among Saudi Adults: Findings from a National Survey, 2013. *J. Community Health* **2015**, *40*, doi:10.1007/s10900-015-0014-4.

84. Al-Shahwan, M.A.; Al-Othman, A.M.; Al-Daghri, N.M.; Sabico, S.B. Effects of 12-month, 2000IU/day vitamin D supplementation on treatment naïve and vitamin D deficient Saudi type 2 diabetic patients. *Saudi Med. J.* **2015**, *36*, doi:10.15537/smj.2015.12.12923.

85. Memish, Z.A.; Chang, J.L.; Saeedi, M.Y.; Al Hamid, M.A.; Abid, O.; Ali, M.K. Screening for Type 2 Diabetes and Dysglycemia in Saudi Arabia: Development and Validation of Risk Scores. *Diabetes Technol. Ther.* **2015**, *17*, doi:10.1089/dia.2014.0267.

86. Zeidan, Z.A.; Sultan, I.E.; Guraya, S.S.; Al-Zalabani, A.H.; Khoshhal, K.I. Low bone mineral density among young healthy adult Saudi women: Prevalence and associated factors in the age group of 20 to 36 years. *Saudi Med. J.* **2016**, *37*, doi:10.15537/smj.2016.11.16248.

87. El Bcheraoui, C.; Tuffaha, M.; Daoud, F.; Kravitz, H.; Al Mazroa, M.A.; Al Saeedi, M.; Memish, Z.A.; Basulaiman, M.; Al Rabeeah, A.A.; Mokdad, A.H. On your mark, get set, go: Levels of physical activity in the Kingdom of Saudi Arabia, 2013. *J. Phys. Act. Heal.* **2016**, *13*, doi:10.1123/jpah.2014-0601.

88. Aboel-Fetoh, N.M.; Alanazi, A.R.; Alanazi, A.S.; Alruwili, A.N. ABO blood groups and risk for obesity in Arar, Northern Saudi Arabia. *J. Egypt. Public Health Assoc.* **2016**, *91*, doi:10.1097/01.EPX.0000508457.31670.20.

89. Asmaa F. Hamouda The Association between Lifestyle, Anthropometric Measurements, and Obesity in University Students. *J. Pharm. Pharmacol.* **2016**, *4*, doi:10.17265/2328-2150/2016.01.003.

90. Alkhalaf, M.M.; Edwards, C.A.; Lean, M.E.J.; Combet, E. Risk classification paradox of anthropometric measurements in Saudi Arabia: need for further consideration. In Proceedings of the Proceedings of the Nutrition Society; 2016; Vol. 75.

91. Mahassni, S.H.; Bashanfar, N.O. Waist Circumference a Predictor of Hypertension and Dyslipidemia in Young Waist Circumference a Predictor of Hypertension and Dyslipidemia in Young Saudi Females. **2016**, *2*, 15–24.

92. Mahmoud, M.; Ncibi, S. Students health status related to their diary intakes: case study in Jazan, Kingdom of Saudi Arabia. *Int. J. Med. Sci. Public Heal.* **2016**, *5*, 1505, doi:10.5455/IJMSPH.2016.01042016415.

93. Brahim, M. Ben; Bougatfa, R.; Abukhaizaran, O.; Moore, P.; Kim, W.D. Body Mass Index: A Comparison of Male Saudi Students to Americans of the Same Age. *Adv. Phys. Educ.* **2016**, *06*, doi:10.4236/ape.2016.64035.

94. Mansour, M.A.A. Developing an anthropometric database for Saudi students and comparing Saudi dimensions relative to Turkish and Iranian peoples. *Am. J. Eng. Appl. Sci.* **2016**, *9*, doi:10.3844/ajeassp.2016.547.557.

95. Abdel-salam, D. Prevalence of Obesity and Its Associated Factors among Female Students at AlJouf University , Saudi Arabia. *Natl. J. Res. Community Med.* **2016**, *5*.

96. Galal Abd El-Aal, B. Studying the Relationship Between Body Mass Index, Waist-Hip Ratio and Quality of Life Among Adult Saudi Females. *Am. J. Nurs. Sci.* **2016**, *5*, doi:10.11648/j.ajns.20160506.13.

97. Bahijri, S.M.; Jambi, H.A.; Al Raddadi, R.M.; Ferns, G.; Tuomilehto, J. The prevalence of diabetes and prediabetes in the adult population of Jeddah, Saudi Arabia - A community-based survey. *PLoS One* **2016**, *11*, doi:10.1371/journal.pone.0152559.

98. Habib, S.R.; Alghofaily, S.; Alshamrani, H.; Alhammad, A.; Awan, K.H. Relationship of body mass index with diet, physical activities, and lifestyles of dental students. *J. Contemp. Dent. Pract.* **2017**, *18*, doi:10.5005/jp-journals-10024-2146.

99. Khabaz, M.N.; Bakarman, M.A.; Baig, M.; Ghabrah, T.M.; Gari, M.A.; Butt, N.S.; Alghanmi, F.; Balubaid, A.; Alzahrani, A.; Hamouh, S. Dietary habits, lifestyle pattern and obesity among young Saudi university students. *J. Pak. Med. Assoc.* **2017**, *67*.

100. Alahmari, K.A.; Silvian, S.P.; Reddy, R.S.; Kakaraparthi, V.N.; Ahmad, I.; Alam, M.M. Hand grip strength determination for healthy males in Saudi Arabia: A study of the relationship with age, body mass index, hand length and forearm circumference using a hand-

held dynamometer. *J. Int. Med. Res.* **2017**, *45*, 540–548, doi:10.1177/0300060516688976.

101. Kaddam, I.M.; Al-Shaikh, A.M.; Abaalkhail, B.A.; Asseri, K.S.; Al-Saleh, Y.M.; Al-Qarni, A.A.; Al-Shuaibi, A.M.; Tamimi, W.G.; Mukhtar, A.M. Prevalence of vitamin D deficiency and its associated factors in three regions of Saudi Arabia: A cross-sectional study. *Saudi Med. J.* **2017**, *38*, doi:10.15537/smj.2017.4.18753.

102. Alghamdi, R.Q. A randomized controlled trial of a 12-week intensive lifestyle intervention program at a primary care obesity clinic for adults in western Saudi Arabia. *Saudi Med. J.* **2017**, *38*, doi:10.15537/smj.2017.8.20553.

103. Abulmeaty, M.M.A.; Almajwal, A.M.; Almadani, N.K.; Aldosari, M.S.; Alnajim, A.A.; Ali, S.B.; Hassan, H.M.; Elkatawy, H.A. Anthropometric and central obesity indices as predictors of long-term cardiometabolic risk among saudi young and middle-aged men and women. *Saudi Med. J.* **2017**, *38*, doi:10.15537/smj.2017.4.18758.

104. Albawardi, N.M.; Jradi, H.; Almalki, A.A.; Al-Hazzaa, H.M. Level of sedentary behavior and its associated factors among Saudi women working in office-based jobs in Saudi Arabia. *Int. J. Environ. Res. Public Health* **2017**, *14*, doi:10.3390/ijerph14060659.

105. Azzeh, F.S.; Bukhari, H.M.; Header, E.A.; Ghabashi, M.A.; Al-Mashi, S.S.; Noorwali, N.M. Trends in overweight or obesity and other anthropometric indices in adults aged 18-60 years in western Saudi Arabia. *Ann. Saudi Med.* **2017**, *37*, doi:10.5144/0256-4947.2017.106.

106. Alqahtani, A.; Aloraini, M.; Alsubaie, A.; Alateq, A.; Alsagabi, B.; Benajiba, N. Comparison of lifestyle patterns and body weight management practices between normal weight and obese female university students (Riyadh – Saudi Arabia). *North African J. Food Nutr. Res.* **2017**, *1*, 11–19, doi:10.51745/najfnr.1.1.11-19.

107. Altowijri, A.; Alloubani, A.; Abdulhafiz, I.; Saleh, A. Impact of nutritional and environmental factors on vitamin D deficiency. *Asian Pacific J. Cancer Prev.* **2018**, *19*, doi:10.22034/APJCP.2018.19.9.2569.

108. Al-Ghamdi, S.; Shubair, M.M.; Aldiab, A.; Al-Zahrani, J.M.; Aldossari, K.K.; Househ, M.; Nooruddin, S.; Razzak, H.A.; El-Metwally, A. Prevalence of overweight and obesity based on the body mass index; A cross-sectional study in Alkharj, Saudi Arabia. *Lipids Health Dis.* **2018**, *17*, doi:10.1186/s12944-018-0778-5.

109. Waleed, B. Anthropometric measurements for young males in Saudi Arabia. *Int. J. Sociol. Anthropol.* **2018**, *10*, doi:10.5897/ijsa2016.0693.

110. Alrashdan, A.; Albassam, M.; Alkohlani, A.; Alkadi, M. Anthropometry of Saudi Arabian male workers. *Proc. Int. Conf. Ind. Eng. Oper. Manag.* **2018**, *2018-March*, 2358–2364.

111. Latif, R.; Rafique, N. Association of anthropometric measurements with oxidant-antioxidant status among young Saudi females. *Physiol. Res.* **2018**, *67*, doi:10.33549/physiolres.933785.

112. Al-Rubeaan, K.; Bawazeer, N.; Al Farsi, Y.; Youssef, A.M.; Al-Yahya, A.A.; AlQumaidi, H.; Al-Malki, B.M.; Naji, K.A.; Al-Shehri, K.; Al Rumaih, F.I. Prevalence of metabolic syndrome in Saudi Arabia - a cross sectional study. *BMC Endocr. Disord.* **2018**, *18*, doi:10.1186/s12902-018-0244-4.

113. Gutierrez, J.; Alloubani, A.; Mari, M.; Alzaatreh, M. Cardiovascular Disease Risk Factors: Hypertension, Diabetes Mellitus and Obesity among Tabuk Citizens in Saudi Arabia. *Open Cardiovasc. Med. J.* **2018**, *12*, doi:10.2174/1874192401812010041.

114. Aldossari, K.K.; Aldiab, A.; Al-Zahrani, J.M.; Al-Ghamdi, S.H.; Abdelrazik, M.; Batais, M.A.; Javad, S.; Nooruddin, S.; Razzak, H.A.; El-Metwally, A. Prevalence of Prediabetes, Diabetes, and Its Associated Risk Factors among Males in Saudi Arabia: A Population-Based Survey. *J. Diabetes Res.* 2018, *2018*.

115. Alsheef, M.; Alassiry, H.; Alotaibi, E.; Alamri, N.; Halwani, A.; Wani, T.; Aljishi, H. Central obesity among adult Saudi males in Riyadh city: Prevalence, risk factors, and associated morbidities. *Saudi J. Obes.* **2018**, *6*, doi:10.4103/sjo.sjo_11_17.

116. Al-Zahrani, J.M.; Aldiab, A.; Aldossari, K.K.; Al-Ghamdi, S.; Batais, M.A.; Javad, S.; Nooruddin, S.; Zahid, N.; Razzak, H.A.; El-Metwally, A. Prevalence of Prediabetes, Diabetes and Its Predictors among Females in Alkharj, Saudi Arabia: A cross-sectional study. *Ann. Glob. Heal.* **2019**, *85*, doi:10.5334/aogh.2467.

117. Almuzaini, Y.; Jradi, H. Correlates and Levels of Physical Activity and Body Mass Index Among Saudi Men Working in Office-Based Jobs. *J. Community Health* **2019**, *44*, doi:10.1007/s10900-019-00639-4.

118. Rafique, N.; AlSheikh, M.H. Identifying menarcheal age and its association with body mass index in young Saudi females. *Saudi Med. J.* **2019**, *40*, doi:10.15537/smj.2019.9.24425.

119. Alquaiz, A.M.; Siddiqui, A.R.; Kazi, A.; Batais, M.A.; Al-Hazmi, A.M. Sedentary lifestyle and Framingham risk scores: A population-based study in Riyadh city, Saudi Arabia. *BMC Cardiovasc. Disord.* **2019**, *19*, doi:10.1186/s12872-019-1048-9.

120. Al-Qahtani, A.M. Prevalence and Predictors of Obesity and Overweight among Adults Visiting Primary Care Settings in the Southwestern Region, Saudi Arabia. *Biomed Res. Int.* **2019**, *2019*, doi:10.1155/2019/8073057.

121. Alamri, E.S. The association between the timing of energy intake and the risk of overweight and obesity among Saudi female university students. *Saudi Med. J.* **2019**, *40*, doi:10.15537/smj.2019.12.24686.

122. Wahabi, H.A.; Fayed, A.A.; Tharkar, S.; Esmaeil, S.A.; Bakhsh, H. Postpartum Weight Retention and Cardiometabolic Risk among Saudi Women: A Follow-Up Study of RAHMA Subcohort. *Biomed Res. Int.* **2019**, *2019*, doi:10.1155/2019/2957429.

123. Alnasser, A.; Kyle, J.; Aloumi, N.; Al-Khalifa, A.; Marais, D. The twazon Arabic weight loss app: App-based intervention for Saudi women with obesity. *JMIR mHealth uHealth* **2019**, *7*, doi:10.2196/10923.

124. Dhaifullah, E.; Al-Maweri, S.A.; Koppolu, P.; Elkhtat, E.; Mostafa, D.; Mahgoub, M. Body mass index and periodontal health status among young Saudi adults: A cross-sectional study. *Ann. Saudi Med.* **2019**, *39*, doi:10.5144/0256-4947.2019.433.

125. Tedla, J.S.; Asiri, F.; Alshahrani, M.S.; Sangadala, D.R.; Gular, K.; Rengaramanujam, K.; Mukherjee, D. Reference values of functional and lateral reach test among the young saudi population: Their psychometric properties and correlation with anthropometric parameters. *Med. Sci. Monit.* **2019**, *25*, doi:10.12659/MSM.916443.

126. Al-Raddadi, R.; Bahijri, S.M.; Jambi, H.A.; Ferns, G.; Tuomilehto, J. The prevalence of obesity and overweight, associated demographic and lifestyle factors, and health status in the adult population of Jeddah, Saudi Arabia. *Ther. Adv. Chronic Dis.* **2019**, *10*, doi:10.1177/2040622319878997.

127. Albassam, R.S.; Lei, K.Y.; Alnaami, A.M.; Al-Daghri, N.M. Correlations of neck circumference with body composition and cardiometabolic risk factors in Arab women. *Eat. Weight Disord.* **2019**, *24*, doi:10.1007/s40519-018-0630-y.

128. Binobead, M.A.; Al-Qahtani, W.H.; Bader, N.A. Al; Alsedairy, S.A.; Arzoo, S. Prevalence of Vitamin D deficiency and the effect of anthropometric and lifestyle factors on theVitamin D statuses of healthy women residing in Riyadh. *Prog. Nutr.* **2019**, *21*, doi:10.23751/pn.v21i2.7105.

129. ElSawy, N.A.; Header, E.A.; Al-Kushi, A.G.; Wahba, H.M.; Bukhari, H.M. Bone mineral density and body composition in Saudi adult females. *MOJ Anat. Physiol.* **2019**, *6*, doi:10.15406/mojap.2019.06.00266.

130. Farhat, K.H.; Arafa, M.A.; Rabah, D.M.; Amin, H.S.; Ibrahim, N.K. Vitamin D status and its correlates in Saudi male population. *BMC Public Health* **2019**, *19*, doi:10.1186/s12889-019-6527-5.

131. El-Metwally, A.; Shaikh, Q.; Aldiab, A.; Al-Zahrani, J.; Al-Ghamdi, S.; Alrasheed, A.A.; Househ, M.; Da'ar, O.B.; Nooruddin, S.; Razzak, H.A.; et al. The prevalence of chronic pain and its associated factors among Saudi Al-Kharj population; A cross sectional study. *BMC Musculoskelet. Disord.* **2019**, *20*, doi:10.1186/s12891-019-2555-7.

132. AlFaris, N.; AlKehayez, N.; AlMushawah, F.; AlNaeem, A.; AlAmri, N.; AlMudawah, E. Anthropometry, bone mineral density and risk of breast cancer in premenopausal and postmenopausal Saudi women. *Arch. Med. Sci.* **2020**, doi:10.5114/aoms.2020.98676.

133. Alkahtani, S.; Aljuhani, O.; Alhussain, M.; Habib, S.S. Association between physical activity patterns and sarcopenia in Arab men. *J. Int. Med. Res.* **2020**, *48*, doi:10.1177/0300060520918694.

134. Amer, O.E.; Sabico, S.; Alfawaz, H.A.; Aljohani, N.; Hussain, S.D.; Alnaami, A.M.; Wani, K.; Al-Daghri, N.M. Reversal of prediabetes in saudi adults: Results from an 18 month lifestyle intervention. *Nutrients* **2020**, *12*, doi:10.3390/nu12030804.

135. Al-Hanawi, M.K.; Chirwa, G.C.; Pemba, L.A.; Qattan, A.M.N. Does prolonged television viewing affect Body Mass Index? A case of the Kingdom of Saudi Arabia. *PLoS One* **2020**, *15*, doi:10.1371/journal.pone.0228321.

136. Syed, N.K.; Syed, M.H.; Meraya, A.M.; Albarraq, A.A.; Al-Kasim, M.A.; Alqahtani, S.; Makeen, H.A.; Yasmeen, A.; Banji, O.J.F.; Elnaem, M.H. The association of dietary behaviors and practices with overweight and obesity parameters among Saudi university students. *PLoS One* **2020**, *15*, doi:10.1371/journal.pone.0238458.

137. Thomas, J.T.; Thomas, T.; Ahmed, M.; Karthiga Kannan, S.; Abdullah, Z.; Alghamdi, S.A.; Joseph, B. Prevalence of periodontal disease among obese young adult population in Saudi Arabia—A cross-sectional study. *Med.* **2020**, *56*, doi:10.3390/medicina56040197.

138. Mumena, W.A.; Alamri, A.A.; Mahrous, A.A.; Alharbi, B.M.; Almohaimeed, J.S.; Hakeem, M.I.; Kutbi, H.A. Knowledge, attitudes, and practices toward added sugar consumption among female undergraduate students in Madinah, Saudi Arabia: A cross-sectional study. *Nutrition* **2020**, *79–80*, doi:10.1016/j.nut.2020.110936.

139. Shaaban, E.; Ali, M. A comparative Study of Anthropometric Variables among Egyptian and Saudi Arabia People. *J. Hum. Sci. – Univ. Cent. Ali Kafi Tindouf - Alger.* **2020**, *4*, 178–190.

140. Mosli, H.H.; Kutbi, H.A.; Alhasan, A.H.; Mosli, R.H. Understanding the Interrelationship between Education, Income, and Obesity among Adults in Saudi Arabia. *Obes. Facts* **2020**, *13*, doi:10.1159/000505246.

141. Naguib, R.; Tawfik, M.R.; Alsubaiei, S.; Almoallem, A.; Alajlouni, D.; Alruwaili, T.; Sendy, W.; Al Habib, Z. Study of bodyweight and eating attitude among female university members in the Kingdom of Saudi Arabia: A comparison between different methods of weight assessment. *J. Fam. Med. Prim. Care* **2020**, *9*, doi:10.4103/jfmpc.jfmpc_1058_19.

142. Toriola, O.O.; Onagbiye, S.O. Body image, weight discrepancy and body mass index among university students in Riyadh, Saudi Arabia. *African J. Phys. Act. Heal. Sci.* **2020**, *26*, doi:10.37597/ajphes.2020.26.4.7.

143. Alahmari, K.A.; Rengaramanujam, K.; Reddy, R.S.; Samuel, P.S.; Kakaraparthi, V.N.; Ahmad, I.; Tedla, J.S. Cardiorespiratory

Fitness as a Correlate of Cardiovascular, Anthropometric, and Physical Risk Factors: Using the Ruffier Test as a Template. *Can. Respir. J.* **2020**, *2020*, doi:10.1155/2020/3407345.

144. Alzeidan, R.; Fayed, A.; Rabiee, F.; Hersi, A.; Elmorshedy, H. Diagnostic performance of waist-to-height ratio in identifying cardiovascular risk factors and metabolic syndrome among adult Saudis. *Saudi Med. J.* **2020**, *41*, doi:10.15537/smj.2020.3.24915.

145. Muaidi, Q.I.; Ahsan, M. Gender-Specific Associations of Different Anthropometric Indices with Sleep Quality and Daytime Sleepiness. *Open Public Health J.* **2020**, *13*, doi:10.2174/1874944502013010273.

146. Sami, R.; Bushnaq, T.; Radhi, K.; Benajiba, N.; Helal, M. PREVALENCE OF THINNESS CASES AND DIETARY DIVERSITY AMONG LEARNERS OF VARIOUS EDUCATION STAGES IN TAIF REGION, SAUDI ARABIA. *African J. Food, Agric. Nutr. Dev.* **2020**, *20*, doi:10.18697/ajfand.95.19520.

147. Aldossari, K.K.; Shubair, M.M.; Al-Zahrani, J.; Alduraywish, A.A.; Alahmary, K.; Bahkali, S.; Aloudah, S.M.; Almustanyir, S.; Al-Rizqi, L.; El-Zahaby, S.A.; et al. Association between Chronic Pain and Diabetes/Prediabetes: A Population-Based Cross-Sectional Survey in Saudi Arabia. *Pain Res. Manag.* **2020**, *2020*, doi:10.1155/2020/8239474.

148. Alshamiri, M.Q.; Mohd A Habbab, F.; Al-Qahtani, S.S.; Alghalayini, K.A.; Al-Qattan, O.M.; El-Shaer, F. Waist-to-Height Ratio (WHtR) in Predicting Coronary Artery Disease Compared to Body Mass Index and Waist Circumference in a Single Center from Saudi Arabia. *Cardiol. Res. Pract.* **2020**, *2020*, doi:10.1155/2020/4250793.

149. Bahijri, S.M.; Ajabnoor, G.M.; Hegazy, G.A.F.; Borai, A.A.; Eldakhakhny, B.M.; Alsheikh, L.N.; Harakeh, S.M. Diet influences levels of plasma lipopolysaccharide (LPS) and its soluble receptor (sCD14) in Saudis. *J. Pak. Med. Assoc.* **2020**, *70*, doi:10.5455/JPMA.28279.

150. Al-Musharaf, S.; McTernan, P.G.; Hussain, S.D.; Aleisa, K.A.; Alnaami, A.M.; Wani, K.; Saravanan, P.; Al-Daghri, N. Prevalence and indicators of vitamin b12 insufficiency among young women of childbearing age. *Int. J. Environ. Res. Public Health* **2021**, *18*, doi:10.3390/ijerph18010001.

151. Al-Musharaf, S.; Aljuraiban, G.; Bogis, R.; Alnafisah, R.; Aldhwayan, M.; Tahrani, A. Lifestyle changes associated with COVID-19 quarantine among young Saudi women: A prospective study. *PLoS One* 2021, *16*.

152. Al-Musharaf, S.; Alabdulaaly, A.; Mujalli, H. Bin; Alshehri, H.; Alajaji, H.; Bogis, R.; Alnafisah, R.; Alfehaid, S.; Alhodaib, H.; Murphy, A.; et al. Sleep quality is associated with vitamin b12 status in female arab students. *Int. J. Environ. Res. Public Health* **2021**, *18*, doi:10.3390/ijerph18094548.

153. Alquaiz, A.M.; Kazi, A.; Almigbal, T.H.; Alhazmi, A.M.; Qureshi, R.; Alhabeeb, K.M. Factors associated with an unhealthy lifestyle among adults in Riyadh City, Saudi Arabia. *Healthc.* **2021**, *9*, doi:10.3390/healthcare9020221.

154. Aldossari, K.K.; Shubair, M.M.; Al-Ghamdi, S.; Al-Zahrani, J.; AlAjmi, M.; Mastour Alshahrani, S.; Alsalamah, M.; Al-Khateeb, B.F.; Bahkali, S.; El-Metwally, A. The association between overweight/obesity and psychological distress: A population based cross-sectional study in Saudi Arabia. *Saudi J. Biol. Sci.* **2021**, *28*, doi:10.1016/j.sjbs.2021.02.008.

155. Abolfotouh, M.A.; Daffallah, A.A.; Khan, M.Y.; Khattab, M.S.; Abdulmoneim, I. Central obesity in elderly individuals in south-western Saudi Arabia: Prevalence and associated morbidity. *East. Mediterr. Heal. J.* **2001**, *7*, doi:10.26719/2001.7.4-5.716.

156. Al-Othaimeen, A.I.; Al-Nozha, M.; Osman, A.K. Obesity: An emerging problem in Saudi Arabia. Analysis of data from the national nutrition survey. *East. Mediterr. Heal. J.* **2007**, *13*.

157. Alzahrani, S.H.; Abdelmoneim El Sayed, I.; Alshamrani, S.M. Prevalence and factors associated with geriatric malnutrition in an outpatient clinic of a teaching hospital in Jeddah, Saudi Arabia. *Ann. Saudi Med.* **2016**, *36*, doi:10.5144/0256-4947.2016.346.

158. Alhamdan, A.; Al-Muammar, M.; Bindawas, S.; Alshammari, S.; Al-Amoud, M.; Calder, P. Body composition analysis by bioelectrical impedance and its relationship with nutritional status in older adults: a cross-sectional descriptive study. *Prog. Nutr.* **2020**.
